# Hemopexin promotes angiogenesis via up-regulating HO-1 in rats after cerebral ischemia-reperfusion injury

**DOI:** 10.1186/s12871-017-0466-4

**Published:** 2018-01-03

**Authors:** Beibei Dong, Zhishen Zhang, Keliang Xie, Yongyan Yang, Yuan Shi, Chenxu Wang, Yonghao Yu

**Affiliations:** Department of Anesthesiology, Tianjin Institute of Anesthesiology, General Hospital of Tianjin Medical University, No. 154 Anshan Road, Heping District, Tianjin, 300052 People’s Republic of China

**Keywords:** Cerebral ischemia, Reperfusion injury, Hemopexin, Heme oxygenase, Neovascularization

## Abstract

**Background:**

Ischemia-reperfusion (I/R) is a critical pathophysiological change of ischemic stroke. Heme-oxygenase-1 (HO-1) is a rate-limiting enzyme of eliminating excessive free heme by combining with hemopexin (HPX), a plasma protein contributing to alleviating infarct size due to ischemia stroke. This study was to investigate whether HPX could improve angiogenesis after cerebral ischemia-reperfusion via up-regulating HO-1.

**Methods:**

Rats were randomly divided into five groups: sham, MCAO, MCAO + Vehicle, MCAO + HPX and MCAO + HPX + protoporphyrin IX (ZnPPIX, an HO-1 inhibitor). Cerebral I/R was induced by MCAO. Saline, vehicle, HPX and HPX + ZnPPIX were respectively given to MCAO group, MCAO + Vehicle group, MCAO + HPX group and MCAO + HPX + ZnPPIX group at the moment after reperfusion by intracerebroventricular injection. Neurological behavioral scores(NBS) was assessed at 24 h and 7d after I/R. Real-time polymerase chain reaction (RT-PCR) was used to analyze the mRNA level of HO-1. Angiogenesis in penumbra area was assessed by immunofluorescence detection at 7d after I/R. Serum endothelial nitric oxide synthase (eNOS) was assessed by enzyme linked immunosorbent assay (ELISA) at 24 h and 7d after I/R.

**Results:**

Compared with sham group, the NBS and the mRNA levels of HO-1 at 24 h and 7d after I/R in MCAO group decreased notably (*P* < 0.05), the new vessel density in ischemia penumbra increased notably at 7d after I/R (*P* < 0.05), the serum eNOS level increased at 24 h and 7d after I/R (*P* < 0.05). MCAO group and MCAO + Vehicle group showed no significant differences (*P* > 0.05). In the MCAO + HPX group, compared with MCAO + Vehicle group, the NBS and the mRNA levels of HO-1 increased drastically at 24 h and 7d after I/R (*P* < 0.05), the new vessel density in ischemia penumbra increased significantly at 7d after I/R (*P* < 0.05), the serum eNOS level at 24 h and 7d after I/R ascended notably (*P* < 0.05). Compared with MCAO + HPX group, the NBS assessment, new vessel density and serum eNOS level decreased at corresponding time points after I/R in MCAO + HPX+ ZnPPIX group (*P* < 0.05).

**Conclusion:**

HPX can promote angiogenesis after cerebral ischemia-reperfusion injury in rats via up-regulating HO-1.

## Background

Ischemia-reperfusion (I/R) is a critically pathophysiological change of ischemia stroke. Mechanisms of I/R injury are related to oxidative stress [1], apoptosis [2], mitochondrial dysfunction [3], etc. A number of therapeutic strategies have been developed over the past decades. However, the effects of the interventions were far from satisfactory [4].

Studies have shown that endothelial injury and dysfunction might be the crucial initiating process of cerebral ischemia [5]. Improvement of the vascular endothelial function is closely related to recovery of neurological function [6]. Excessive free heme is highly cytotoxic which would cause endothelial injury [7]. Hemopexin (HPX) is able to remove excess free heme, Heme-oxygenase-1 (HO-1) is rate-limiting enzyme [8]. Our previous animal studies showed the expression of HPX in neurons of central nervous system and brain vascular system. Intraventricular injection of HPX could reduce the ischemic infarct volume of cerebral rats [9]. However, neuroprotective effect of HPX and its mechanism are poorly studied, so we designed experiments to explore whether HPX could provide any benefit on angiogenesis after cerebral ischemia-reperfusion injury, thus providing a new therapy to promote the recovery of cognitive function after stroke.

## Methods

### Animals

Male Sprague–Dawley (SD) rats, 7–8 weeks old, weighing 250-280 g, were purchased from the experimental animal center of Chinese Military Medical Science Academy of the PLA(license number: SCXK_ (Army) 2009–003). Animals were housed in specific pathogen free environment: temperature 22 ± 2 °C, relative humidity of about 55–60%, 12 h daily alternating day/night, free access to food and drinking water. 120 SD rats were divided into 5 groups: Sham, middle cerebral artery occlusion (MCAO), MCAO + Vehicle, MCAO + HPX, MCAO + HPX+ protoporphyrin IX (ZnPPIX, *n* = 24 per group). This study has been approved by Medical University of Tianjin experimental animal management committee (Aecl2015–0158 [JIN]; October 27, 2015).

### Cerebral ischemia reperfusion model

MCAO model was used to establish the rat model of middle cerebral artery occlusion by suture method, as previously described [10]. Anesthesia was induced by intraperitoneal injection of 10% chloral hydrate (0.3 g/kg). Right common carotid artery (CCA), right external carotid artery (ECA) and carotid artery bifurcation were exposed and separated. Small opening was cut with ophthalmic scissors on CCA at the distance of 5 to 15 mm from artery bifurcation. Monofilament nylon suture (purchased from Beijing seino Biotechnology Co. Ltd) was inserted inward into the intracranial through CCA until slightly resistance was felt. After 2 h of ischemia, the suture was withdrawn and the wound was stitched. After the rats were awake, the behavior of rats was evaluated using the method of Longa [11], the model was deemed successful when the score was between 1 and 3. Rats failed to reach this standard were ruled out in the following experiments. Rats in the sham operation group were exposed under the same conditions. Right CCA, right ECA and ligation of the right carotid bifurcation was exposed without suture inserted. Electric blanket was used in the process to maintain the temperature of the rats at 37 + 0.5 °C.

### Treatment

Rats (*n* = 6) were fixed on a stereotaxic apparatus immediately after I/R. Saline (10 μL, 0.9%), sodium azide (NaN_3_, 10 μL, 0.1%), HPX (10 μL, 1.86 g/L, ICL, USA, Lot: RS-25HX) and HPX + ZnPPIX (Cayman, USA, Lot:16375) mixture (10 μL) were injected intracerebroventricularly according to the previous study [12].

### Neurobehavioral evaluation

Garcia score method was adopted by a researcher blinded to the groups of this study. The neurobehavioral evaluation was done through the neurological behavioral score (NBS) [13]. The score was divided into two evaluations of motor function and sensory function, with the highest 18 points and a minimum 3 points. The higher score represents the better neural behavior: score 3–7 showed severe neurological dysfunction, 8–11 moderate, and 12–18 were divided into mild neurological dysfunction.

### Real time PCR

Real time PCR was used to detect the change of HO-1 mRNA level in ischemic penumbra. Anesthesia was induced by intraperitoneal injection of 10% chloral hydrate (0.3 g/kg). Rats of each group (*n* = 6) were then perfused through left ventricular until the liver seemed white and the liquid outflow from the right atrial became clear. Rats were sacrificed by cervical dislocation after prior anesthesia and the ischemic penumbra was obtained [14]. Briefly, the middle coronal brain section was used for determining the ischemic penumbra. A 2 mm longitudinal cut (from top to bottom) was made approximately from the lateral of sagittal suture through right hemisphere followed by a transverse diagonal cut at approximately the “2 o’clock” position to separate the core from the penumbra. Total RNA was extracted and reverse transcribed to cDNA, real-time fluorescence quantitative PCR was detected by the SYBR Green method (GAPDH as internal control). HO-1 primer was77bp in length, the upstream primer: 5 ′-CGACAGCATGTCCCAGGATT-3′, the downstream primer 5 ′-TCGCTCTATCTCCTCTTCCAGG-3′. GAPDH primer was 95 bp in length, upstream primer: 5′-CAGTGCCAGCCTCGTCTCAT-3, the downstream primer 5′-AGGGGCCATCCACAGTCTTC-3′. Amplification and data analysis was done by iQTM multiplex real-time fluorescence quantitative PCR instrument (Bio-Rad, USA). The relative expression of mRNA was calculated by the 2-(△△Ct) method.

### Immunohistochemical staining (IHC)

IHC was used to detect the blood vessel density in rat ischemic penumbra. After anaesthetized by chloral hydrate, the rats were perfused as before. The rat brain tissue was taken, and was frozen and sliced in coronary. Sections were blocked in 10% normal goat serum (dissolved in PBS) supplemented for 1 h at room temperature. Wash with PBS for 5 min. CD31 antibody (1:100, Abcam, UK, ab119339 Lot: GR264335–1) and vWF (von Willebrand factor) antibody (1:100, Abcam, UK, ab6994 Lot: GR223933–3) were then blended and added to the organization, overnight at 4 °C. Sections were washed 3 times with PBS, 5 min each time on the next day. FITC labeled anti mice secondary antibodies (1:100, Chinese fir in jinqiao, Chinese, ZF-0312 Lot:112242) and TR - TC labeled anti rabbit secondary antibodies (1:100, Chinese fir in jinqiao, Chinese, oZF-0317 Lot: 116128) were blended and added to the tissue, 37 °C for 1 h incubation. Wash with PBS for three times, 5 min each time. Sections were incubated with DAPI (1 ng/μl; Sigma-Aldrich, Poole, UK, Lot:D9542) to stain the nuclei for 2 min at room temperature and then washed with PBS for 5 min. Fluorescent signals were detected by confocal laser scanning microscopy (Olympus, FV1000). Observation was done at lower magnification firstly, and then three target area were selected, switch the lens to the high magnification (200 times) and calculate the number of blood vessels in each region. The blood vessel density was represented by the mean blood vessel count at each 200 times magnification of view.

### Endothelial nitric oxide synthase (eNOS) level determination

At 24 h and 7d after I/R, anesthesia was induced by intraperitoneal injection of 10% chloral hydrate, and 3 cm capillary suction which was disinfected in advance was rotated and inserted into the angular, while angular vein plexus blood flowed out. 5 ml peripheral blood sample was taken into the anticoagulant tube. Gently rotated the capillary out and apply pressure to stop bleeding by a cotton ball immediately. Peripheral blood of rats in sham group was obtained in the same way at the corresponding time point as a control. Place enzyme linked immunosorbent assay (ELISA) reagent (Nanjing jiancheng institute of Bioengineering, Chinese, Lot: H195) under the temperature of 25 °C for 30 min, then configure the standard at the concentration of 10.0, 5.0, 2.5, 1.0, 0.5 and 0.0 ng/mL, add 100 ul to each hole in order. 100 ul serum samples was added in the blank hole, 100 ul distilled water was added in the blank control hole. 50 ul enzyme marked solution was added in all the holes (excluding blank control group), the reagents were mixed away from light at 37 °C followed by 15 min reaction, and then 50 ul terminated liquid was added into the holes. The OD value and the concentration are read by ELIASA.

### Statistical analyses

Statistical analyses were completed by the SPSS 18.0 data analysis software program. Measurement data were described as mean ± SD, and were analyzed by one-way ANOVA. Differences between groups were detected by Tukey’s post-hoc test. *P* < 0.05 was defined as statistical significance.

## Results

### Neurobehavioral evaluation (NBS)

**Fig. 1:**
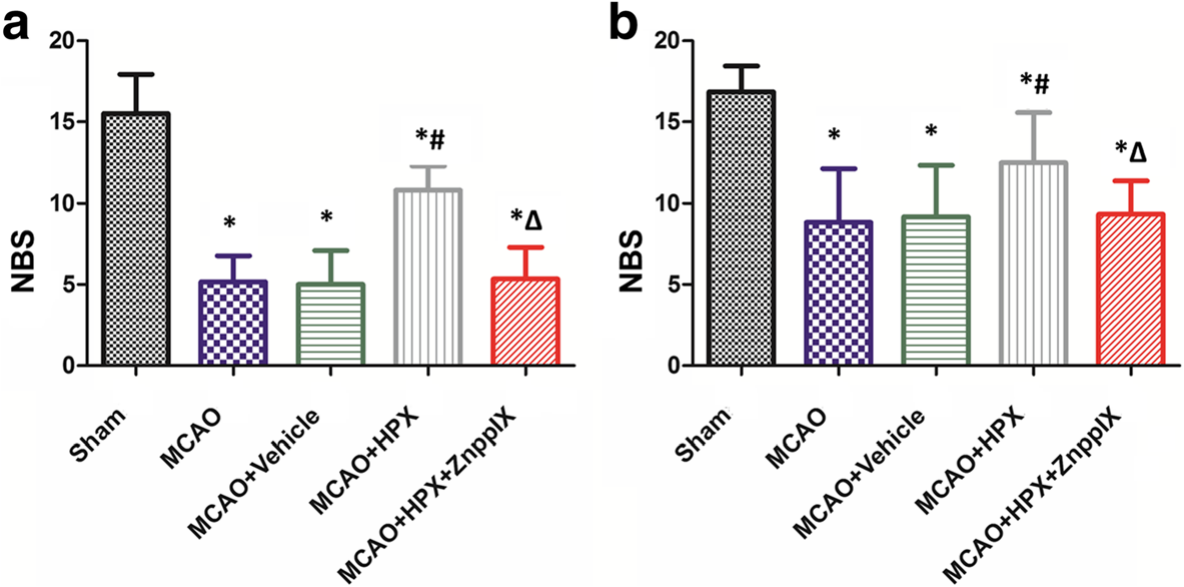
Comparison of NBS scores in the five groups of rats after I/R. **a** Neurobehavioral scores of the rats in five groups at 24 h after I/R; **b** Neurobehavioral scores of the rats in five groups at 7d after I/R; * *P* < 0.05 in comparison with sham group; # *P* *<* 0.05 in comparison with MCAO + Vehicle group; Δ *P* < 0.05 in comparison with MCAO + HPX group

The neurological behavioral score scores (NBS) of rats in each group at 24 h after I/R were 15.5 ± 2.43 (sham), 5.17 ± 1.60 (MCAO), 5.00 ± 2.10 (MCAO + Vehicle), 10.83 ± 1.47 (MCAO + HPX), and 5.33 ± 1.97 (MCAO + HPX + ZnPPIX) respectively (*n* = 6). The differences were statistically significant (F = 34.819, *P* < 0.05).At 7d after I/R, the NBS scores of each group were 16.83 ± 1.60, 8.83 ± 3.31, 9.17 ± 3.19, 12.50 ± 3.08, and 9.33 ± 2.07 respectively. The difference was statistically significant (F = 13.113, *P* < 0.05). NBS scores in MCAO group were significantly lower at 24 h and 7d after I/R (P < 0.05) in compared with Sham group. Compared to the MCAO group, there was no significant change in the NBS score of 24 h and 7d after I/R in the MCAO + Vehicle group (*P* > 0.05). Compared with MCAO + Vehicle group, the NBS score in MCAO + HPX group was significantly higher at 24 h and 7d after I/R (*P* < 0.05). The NBS scores of MCAO + HPX + ZnPPIX group were significantly lower than that of MCAO + HPX group (*P* < 0.05) (Fig. 1).

**Fig. 2:**
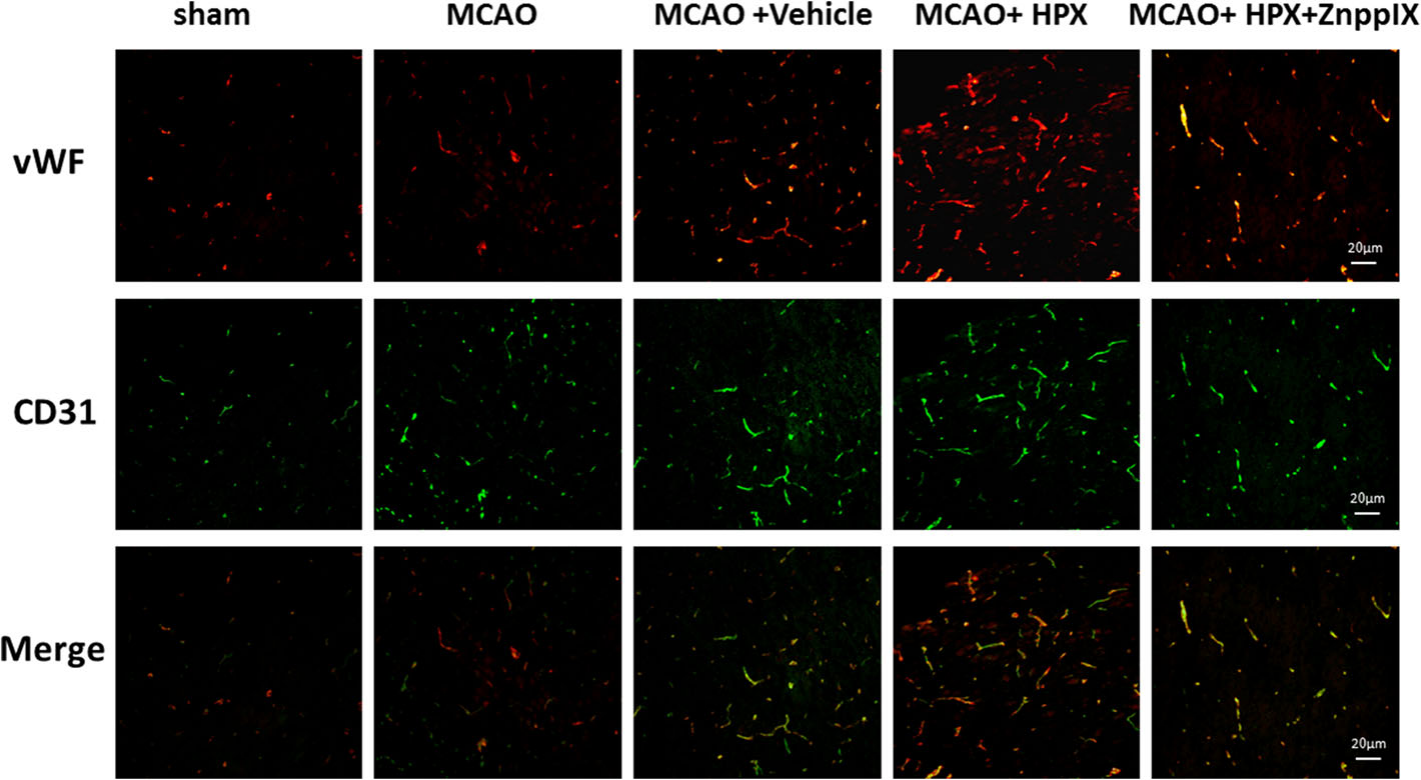
Comparison of blood vessel density in the ischemic penumbra at 7d after I/R. Compared with sham group, the new vessel density of ischemia penumbra in rats of MCAO group rose respectively at 7 d after focal cerebral I/R injury; Compared with Vehicle group, the new vessel density of ischemia penumbra in rats of HPX group saw a marked increase respectively at 7 d after focal cerebral I/R injury; When the inhibitor of HO-1, ZnPPIX, was given along with HPX simultaneously to rats after focal cerebral I/R injury, the positive effect of HPX on enhancing the neovascularization of ischemia penumbra was significantly blocked respectively. However, there was no statistical difference in terms of the new vessel density of ischemia penumbra in rats between MCAO and MCAO + Vehicle group at 7 d after focal cerebral I/R injury respectively. Scale bars = 20 μm

### HO-1 mRNA level in ischemic penumbra

Compared with Sham group, the level of HO-1 mRNA in the ischemic penumbra of cerebral tissue was significantly increased (*P* < 0.05) in the MCAO group rats at 24 h and 7d after I/R. Compared with MCAO group, there was no significant change in the level of HO-1 mRNA in MCAO + Vehicle group at 24 h and 7d (*P* > 0.05). Compared with MCAO + Vehicle group rats, HO-1 mRNA level increased markedly in MCAO + HPX group at 24 h and 7d after I/R (*P* < 0.05) (Table 1).

**Table 1:** Comparison of HO-1 mRNA levels in the four groups at 24 h and 7d after I/R

Groups	N	24 h (arbitrary units)	7d (arbitrary units)
Sham	6	2.16 ± 0.85	1.08 ± 0.14
MCAO	6	5.33 ± 1.45^a^	3.28 ± 1.07^a^
MCAO + Vehicle	6	5.05 ± 1.64^a^	3.16 ± 1.15^a^
MCAO + HPX	6	7.81 ± 1.76^ab^	8.53 ± 2.01^ab^
*F*		14.843	37.240
*P*		0.000	0.000

^a^P < 0.05 compared with Sham group

^b^P < 0.05 compared with MCAO + Vehicle group

### Blood vessel density in ischemic penumbra

The new blood vessel density in the ischemic penumbra at 7d after I/R was1.5 ± 0.75 (sham), 4.17 ± 1.38 (MCAO), 4.50 ± 1.29 (MCAO + Vehicle), 9.17 ± 2.57 (MCAO + HPX), and 5.00 ± 2.28 (MCAO + HPX + ZnPPIX) respectively (The number was obtained by counting every 200 times magnificational view). The difference was statistically significant (F = 13.183, *P* < 0.05). Compared with Sham group, the new blood vessel density in the ischemic penumbra was increased (*P* < 0.05) in MCAO group. Compared with MCAO group, there was no significant change in the new blood vessel density in the ischemic penumbra (*P* > 0.05) in MCAO + Vehicle group. Compared with MCAO + Vehicle group, the MCAO + HPX group had a significant increase in new blood vessel density (*P* < 0.05); Compared with MCAO + HPX group, new blood vessel density of MCAO + HPX + ZnPPIX group decreased markedly in the ischemic penumbra. (Fig 2).

### eNOS level in serum

Compared with Sham group, the level of eNOS in the serum of the rats in MCAO group was increased at 24 h and 7d after I/R (*P* < 0.05). Compared with MCAO group, there was no significant change in the serum eNOS level than in MCAO + Vehicle group (*P* > 0 0.05). Compared with MCAO + Vehicle group rats, the levels of eNOS in the serum of MCAO + HPX group were significantly higher at 24 h and 7d after I/R (*P* < 0.05). Compared with MCAO + HPX group, the levels of eNOS in the serum of MCAO + HPX + ZnPPIX group were significantly decreased (*P* < 0.05) at 24 h and 7d after I/R (Table 2).

**Table 2:** The eNOS levels in serum at 24 h and 7d after I/R

Groups	N	24 h (ng/mL)	7d (ng/mL)
Sham	6	2.50 ± 1.22	6.33 ± 1.51
MCAO	6	8.83 ± 3.76^a^	14.17 ± 3.43^a^
MCAO + Vehicle	6	10.83 ± 3.76^a^	15.17 ± 3.97^a^
MCAO + HPX	6	18.67 ± 4.50^ab^	25.17 ± 4.02^ab^
MCAO + HPX + ZnppIX	6	9.83 ± 3.31^ac^	14.83 ± 2.04^ac^
*F*		16.378	26.799
*P*		0.000	0.000

^a^*P* < 0.05 compared with Sham group

^b^*P* < 0.05 compared with MCAO + Vehicle group

^c^*P* < 0.05 compared with MCAO + HPX group

## Discussion

The middle cerebral artery is the direct continuation of internal carotid artery that the most arterial ischemic occlusion occurs here [15]. However, the arteries provide about 80% of brain’s blood supply, and once occlusion occurs, the clinical symptoms are more serious. In the present study we used classic MCAO method to establish the rat ischemia/reperfusion (I/R) injury model, which is consistent with the clinical characteristics of ischemic cerebral stroke. Compared with varieties of other neurobehavioral evaluation methods, Garcia scoring has better correlation to ischemic stroke volume of infarction in rats [16]. We found that neurobehavioral scores(NBS) in ischemia-reperfusion injury(MCAO) rats at 24 h and 7d after I/R were significantly lower than that in the sham group. Intracerebroventricular injection of HPX significantly improved neurological function within 7d after MCAO. When HO-1 inhibitor ZnPPIX was given together with HPX, NBS scores at 24 h and 7d after I/R reduced to the level as MCAO group. Therefore, we demonstrate that HPX can effectively improve neurologic deficits after I/R in rats, and HO-1 may be the key molecule that is related to the neuroprotective effect of HPX.

Free heme has a lipid structure that is mainly used in the synthesis of hemoglobin, cytochrome P450, catalase, etc. [17]. It is also an important factor for regulating gene expression of these proteins. However, increasing studies have found that overloaded free heme was strongly cytotoxic [18, 19]. The toxic effect is most significant in vascular endothelial cells and neurons [20], varieties of tissue damage are accompanied by excessive release of free heme [21]. In addition, free heme also participates in the oxidative stress and inflammation of the vascular endothelial, causing vascular endothelial dysfunction [22]. HPX has the strongest affinity to free heme in plasma, and can remove excessive free heme. Our previous study showed that HPX expression increased in the vascular system after cerebral ischemia, and the intracerebroventricular injection of HPX showed a dose dependence reduce in the cerebral infarction volume of rats after I/R [9]. As an endogenous protective factor, HO-1 is one of the most sensitive indicator of cellular stress, and play a role of protection in a variety of vascular original injuries [23]. Expression of HO-1 was increased when I/R injury occurs. Exogenous import of HO-1 gene carrier significantly increased animal neurobehavioral score significantly, reduced infarct volumes at 24 h after reperfusion [24].

CD31 (platelet endothelial cell adhesion molecule-1) exists in the edge of the early endothelial cells, reflects contact between endothelial cells and induces intracellular signal transmission. vWF, the carrier protein of coagulation factor VII, plays an important role in the process of blood platelet adhering to the subcutaneous tissue. Co-expression of CD31 and vWF were considered to be the specific marker to identify the endothelial cells [25]. Differentiation of endothelial progenitor cells in ischemic penumbra is observed and the density of neovascularization begins to increase at 3d after ischemia reperfusion. This process may be continued until 21 days after ischemia, indicating the persistence of angiogenesis [26]. Detection of indicators of vascular permeability and endothelial function shows that expression of the endothelial nitric oxide synthase (eNOS) which is related to the vascular permeability and vasodilatation function is up-regulated too [27]. In the present research eNOS expression mediated by HO-1 during I/R process was confirmed through the use of ZnPPIX, inhibitor of HO-1. eNOS promoted repairment of vascular endothelial function and played a favorable role in HPX improving neovascularization and neurologic function during I/R.

It was indicated in the present study that, in comparison with sham group, HO-1 mRNA level in ischemic penumbra of MCAO group irritably increased at 24 h and 7d after I/R. The angiogenesis density in the ischemic penumbra and the level of serum eNOS increased dramatically. Intracerebroventricular injection of HPX remarkably elevated HO-1 mRNA level in ischemic penumbra at 24 h and 7d after I/R. Additionally, the new blood vessel density in the ischemic penumbra and the level of serum eNOS increased. When HPX was given together with ZnPPIX, these effects disappeared. In comparison with MCAO + HPX group, the angiogenesis density in the ischemic penumbra and the level of serum eNOS diminished notably. Our observations implied that HPX could significantly increase the new blood vessel density in the ischemic penumbra after I/R, and HO-1 might be the key signal molecule in the process of HPX promoting angiogenesis in the ischemic penumbra after I/R. HPX exhibits promising superiority to be developed as a clinical applicable treatment for stroke therapy. However, several challenges are remained to be conquered before it can be used in clinical practice. HPX is not allowed to cross blood–encephalic barriers easily to perform its neuroprotective effect due to its high molecular weight. Otherwise, low mean concentration of HPX in cerebrospinal fluid would be insufficient to cope with the quantities of heme released during ischemia reperfusion. It is crucial to find a convenient and clinically available deliver ways to introduce HPX to brain cells for clinical application. Moreover, neuroprotective mechanisms of HPX in stroke are still equivocal. Better functional neurobehavioral tests might not just be a result of neovascularization but might be also a result of attenuated cell death [28]. Fully understanding of the potent mechanisms of HPX will provide theoretical foundation to develop the most optimal therapeutic strategies.

## Conclusions

HPX can promote angiogenesis in rats via up-regulating HO-1 after cerebral ischemia-reperfusion injury.

## Abbreviations

CCA: common carotid artery
ECA: external carotid artery
ELISA: enzyme linked immunosorbent assay
eNOS: endothelial nitric oxide synthase
HO-1: oxygenase −1
HPX: hemopexin
I/R: Ischemia-reperfusion
IHC: immunohistochemical staining
MCAO: middle cerebral artery occlusion
NBS: neurological behavioral score
RT-PCR: real-time polymerase chain reaction
vWF: von Willebrand factor
ZnPPIX: protoporphyrin IX
